# Kidney Allograft Salvage by Autotransplantation for a Suspected Infectious Arterial Pseudoaneurysm in a Pediatric Recipient

**DOI:** 10.1155/crit/1186961

**Published:** 2026-11-05

**Authors:** Zachary H. Rollins, Holly R. Caboti-Jones, Daniel H. Newton, Seung D. Lee, Muhammad I. Saeed, David A. Bruno, Amit Sharma

**Affiliations:** ^1^ Department of Surgery Virginia Commonwealth University and School of Medicine Richmond Virginia USA; ^2^ Division of Vascular Surgery Virginia Commonwealth University Richmond Virginia USA vcu.edu; ^3^ Hume-Lee Transplant Center Virginia Commonwealth University Richmond Virginia USA vcu.edu

## Abstract

Transplant renal artery pseudoaneurysm is a rare complication following kidney transplantation. Noninfected pseudoaneurysms are increasingly managed with endovascular intervention; however, infected pseudoaneurysms often require surgical resection or transplant nephrectomy for definitive management. We report the case of a 14‐year‐old female kidney transplant recipient with a neurogenic bladder who developed recurrent UTIs and bacteremia. The patient was diagnosed with a 3.0 × 2.8 cm renal artery pseudoaneurysm. After a course of antibiotics, she underwent resection of the pseudoaneurysm and an involved segment of the right external iliac artery, followed by kidney autotransplantation to a deceased donor iliac artery interposition graft. Final cultures were negative for bacterial or fungal growth. Six years later, the patient remains off dialysis with a serum creatinine of 2.08 mg/dL (eGFR 34 mL/min/1.73m^2^). A review of the literature suggests that, in carefully selected recipients, excision of renal transplant pseudoaneurysms with renal autotransplantation can allow for allograft salvage.

**Key Takeaways**

• First reported case of autotransplant for a suspected infectious renal arterial pseudoaneurysm in a pediatric renal transplant patient.
• Donor interposition homografts can be safely used in the setting of potentially infectious pseudoaneurysms to minimize infectious risk after repair.
• Core tip: Autotransplantation is a potential salvage strategy for kidney transplants with infectious arterial pseudoaneurysms but surgeons should be aware of the risk of blood loss during this challenging procedure.

## 1. Introduction

Renal transplant arterial pseudoaneurysm is a rare complication after kidney transplantation that can result in serious morbidity or even mortality. Pseudoaneurysms have been reported to occur in less than 1% of kidney transplants [1, 2]. Treatment options include observation, endovascular repair, thrombin injection, surgical revision, or even explantation of the transplanted kidney because of potential arterial rupture [2]. Noninfected aneurysms are increasingly being managed with endovascular interventions, such as coiling or stenting, when the anatomy is amenable. Infectious pseudoaneurysms remain a clinical challenge given concerns for ongoing infection and higher rates of rupture, even after endovascular intervention. A potential treatment technique that allows for both renal allograft preservation and definitive aneurysm treatment is aneurysm excision with autotransplantation. This technique has not previously been reported in the pediatric population. Kidney autotransplantation is typically performed on native kidneys for the management of renovascular disease, ureteral stricture, ureteral trauma, and renal neoplasia [3, 4]. We present the case of kidney allograft preservation through autotransplantation after aneurysm resection in a pediatric recipient with high PRA after the development of an infectious pseudoaneurysm. Written informed consent was obtained from the patient for the publication of case details and the use of images using the Wiley consent to publication form and is available for review. This case seeks to highlight both the feasibility and challenges of this rare graft salvage technique in the pediatric population for pseudoaneurysm management.

## 2. Case Presentation

A 14‐year‐old adopted female presented to Virginia Commonwealth University in March 2020 for deceased donor kidney transplantation through the legally regulated organ donation system with informed consent obtained from the appropriate representatives. The patient′s history was notable for lumbosacral myelomeningocele, neurogenic bladder requiring intermittent catheterization, erythroid hypoplasia, and end‐stage renal disease on dialysis. Prior surgeries included bladder augmentation with bilateral ureteral reimplantation and radical right native nephrectomy for nephroblastoma. She had a high panel‐reactive antibody of 96% secondary to her lifetime transfusion requirement. The patient underwent deceased donor kidney allograft transplant into the extraperitoneal right iliac fossa without issue. The ureter was anastomosed using a mucosa‐to‐mucosa extravesicular technique with 6‐0 PDS and a detrusor tunnel was created using 3‐0 chromic. Postoperatively the patient had initial delayed graft function, but went on to have an eGFR of 66.7 mL/min/1.73m^2^. Immunosuppression was maintained on tacrolimus, prednisone, and Cellcept. On postoperative Day 15, she presented with fevers, urinary tract infection, and ESBL *E. coli* bacteremia. She was found to have a 5.5 × 4.7 cm perinephric abscess (adjacent to the vascular anastomosis) positive for *E. coli*. She was treated with pigtail drainage in addition to vancomycin and cefepime and then transitioned to Meropenem for a 17‐day course. On postoperative Day 41, the patient presented with recurrent fevers. Blood cultures were again positive for ESBL *E. coli* and urine cultures positive for *Enterococcus avium* and *Klebsiella pneumoniae*. An updated CT showed near resolution of the previous perinephric abscess. Meropenem was restarted in addition to a short course of ampicillin. On postoperative Day 75, she presented with fever, fluid overload, and acute kidney injury. Computed tomography identified a 3.0 × 2.8 cm pseudoaneurysm at the arterial anastomosis with surrounding inflammatory changes and severe short‐segment stenosis of the renal artery (Figures 1 and 2). On postoperative Day 78, the patient was taken for an angiogram by interventional radiology which confirmed the pseudoaneurysm and noted mild stenosis of the external iliac artery proximal and distal to the aneurysm due to compression. After, a multidisciplinary discussion involving infectious disease, transplant surgery, interventional radiology, vascular surgery, and transplant nephrology reached a consensus that this aneurysm was most likely due to an infected pseudoaneurysm given the proximity of the recent perinephric fluid collection and positive blood cultures. Two options were offered to the patient′s family: transplant nephrectomy or surgical excision of the pseudoaneurysm with autotransplant. In view of her high PRA and the family′s desire to preserve the kidney, we moved forward with excision of the infected pseudoaneurysm and autotransplantation.

**Figure 1:**
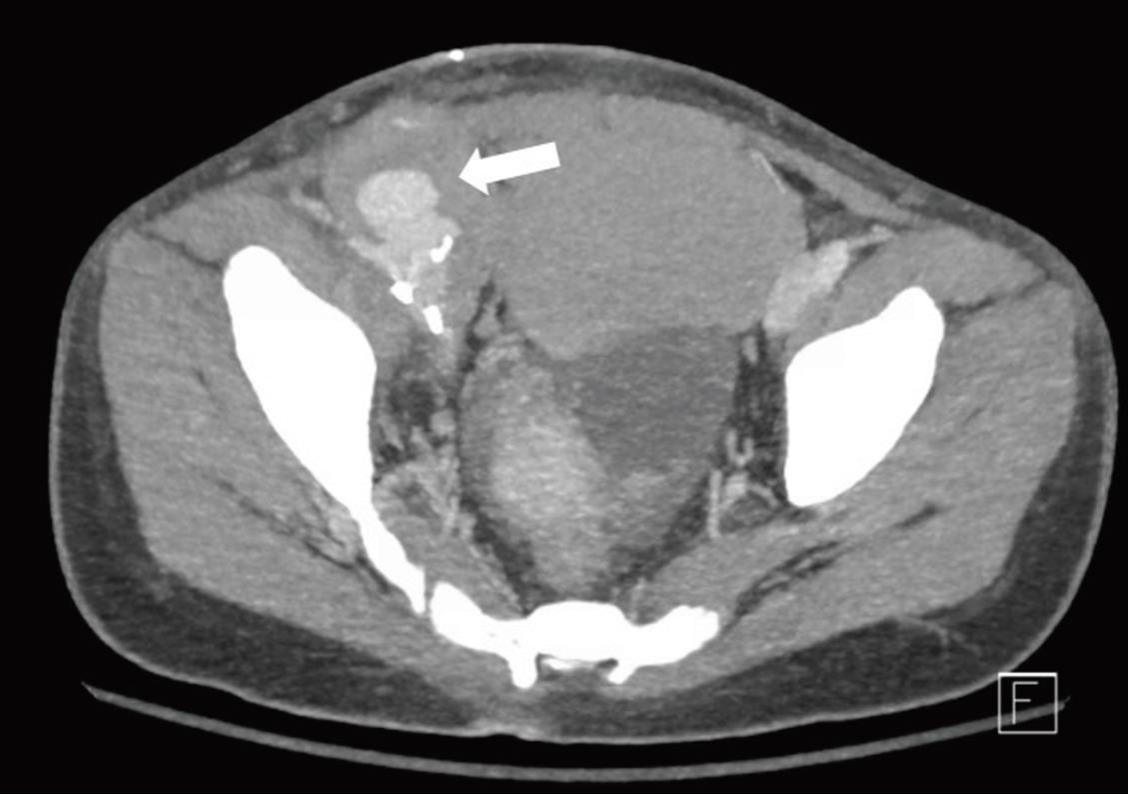
Computed tomography angiogram showing pseudoaneurysm at the site of anastomosis of the renal artery to the right external iliac artery.

**Figure 2:**
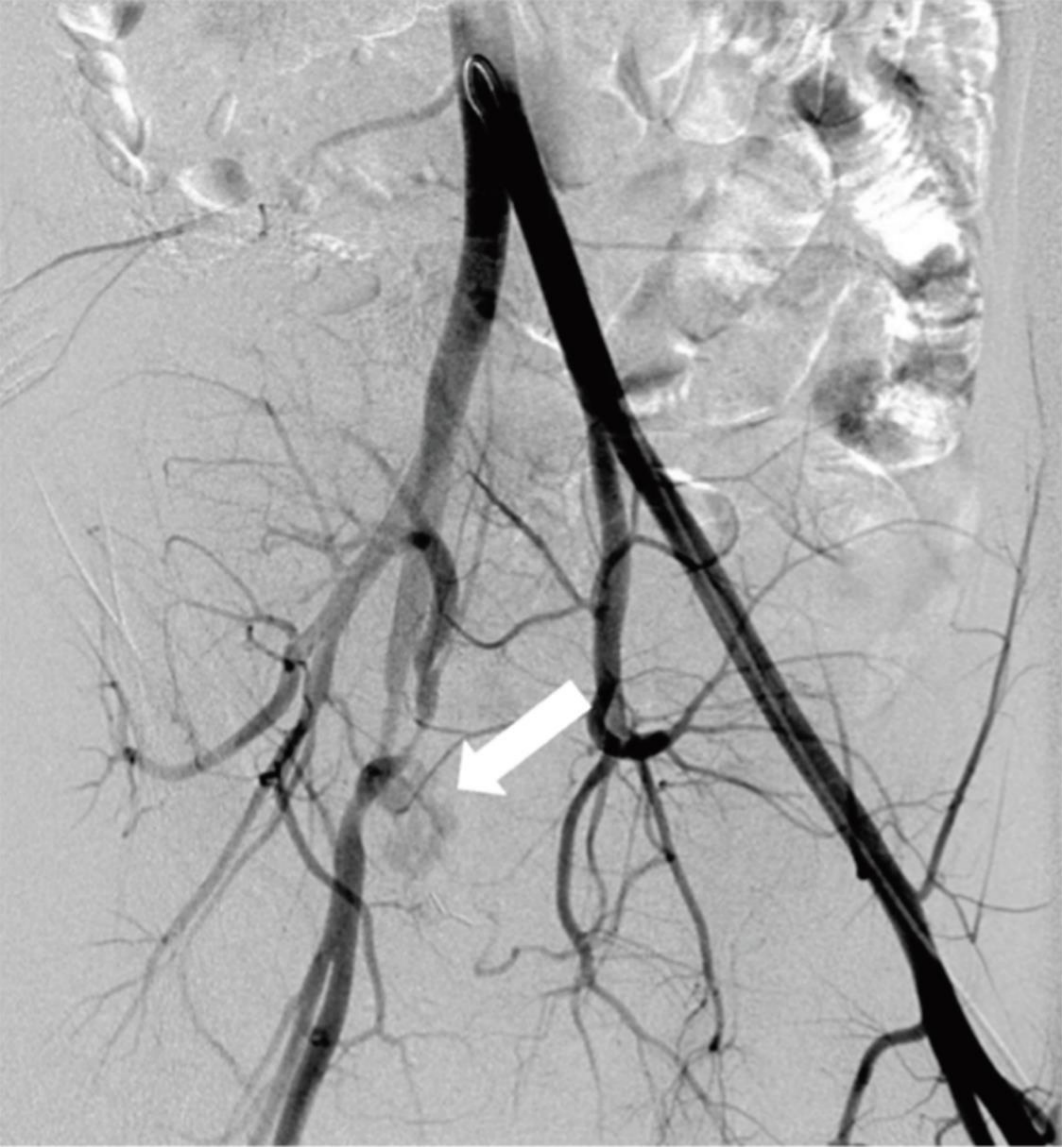
Angiogram showing renal transplant artery pseudoaneurysm.

On postoperative Day 79, the patient was taken back to the operating room. We proceeded with intraperitoneal exposure through a prior midline scar. There were dense fibrotic adhesions in the right iliac fossa. Proximal control of common and internal iliac arteries was obtained. Distal control was achieved by isolation of the common femoral artery. The transplant kidney was completely mobilized to gain access to the distal external iliac artery and the transplant renal artery with pseudoaneurysm. The patient was heparinized and the kidney was explanted by transecting the renal artery and vein proximal to the pseudoaneurysm. We then performed segmental excision of a 6 cm segment of the external iliac artery with the pseudoaneurysm. The pseudoaneurysm was noted to have mural thrombus and was sent for culture. On the back table, the kidney was flushed with cold UW solution and prepared for autotransplantation.

An ABO‐compatible deceased donor external iliac artery from cold storage was tunneled under the inguinal ligament and used to create an end‐to‐side common iliac artery to common femoral arterial bypass with 6‐0 Prolene as an interposition graft. The kidney transplant was then performed to the recipient′s external iliac vein in an end‐to‐side anastomosis with 6‐0 Prolene suture and end‐to‐side arterial anastomosis to the interposition graft with 7‐0 Prolene. The bladder anastomosis was performed to the native bladder using a mucosa‐to‐mucosa extravesicular technique using 6‐0 PDS (Figure 3). Warm ischemic time was 53 min. Despite protamine administration the patient remained coagulopathic. The abdomen was temporarily packed and the patient was transferred to the pediatric intensive care unit. Estimated blood loss was three liters. In total the patient received 10 units of red blood cells, four units of fresh frozen plasma, four platelet transfusions, and one unit of cryoprecipitate.

**Figure 3:**
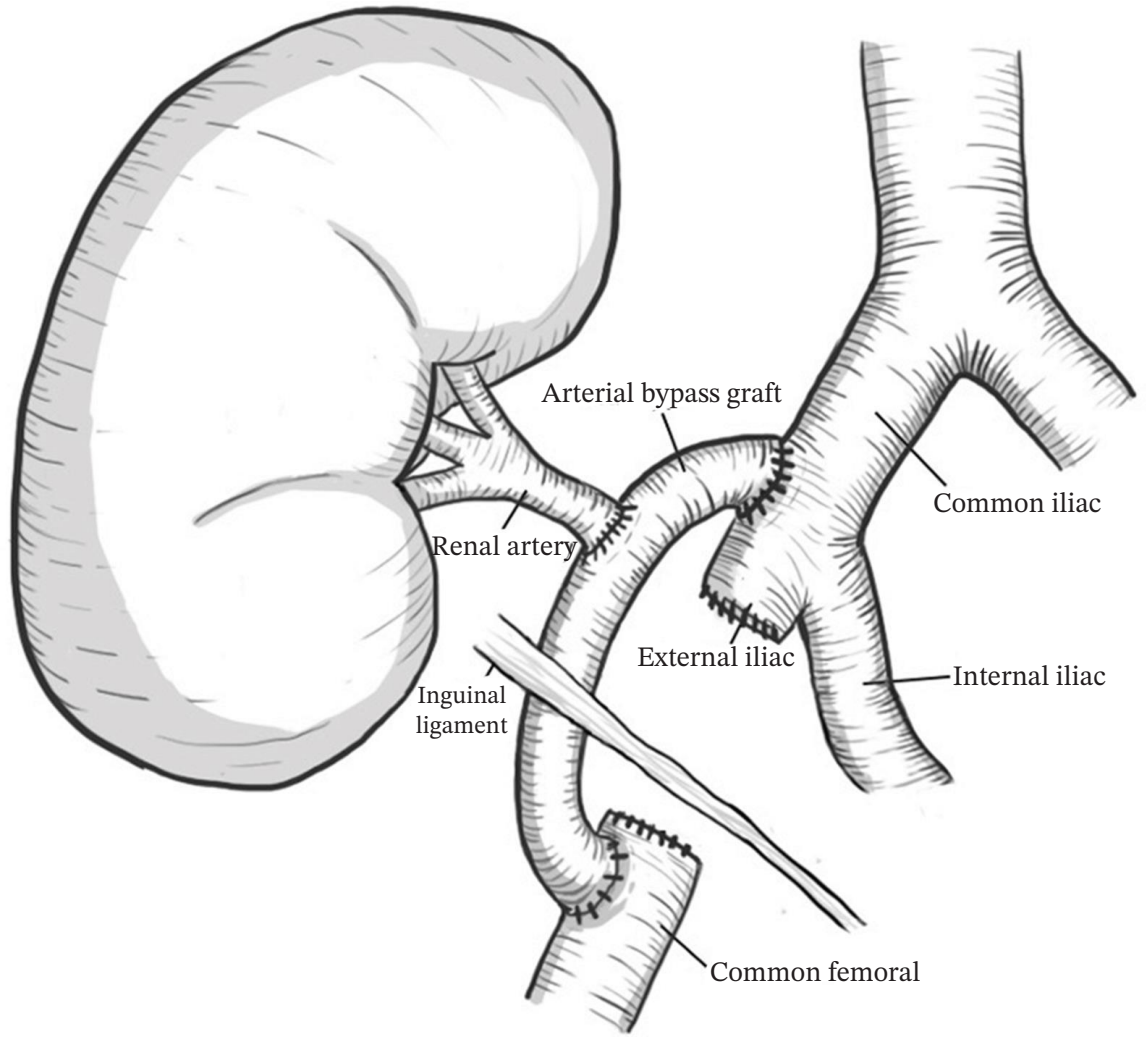
Schematic of autotransplantation of renal allograft to the bypass arterial graft from a deceased donor.

Two days later, the patient was taken for washout and abdominal wound closure. A small urine leak was noted intraoperatively, likely because of ureteral ischemia. The ureteric anastomosis was taken down and the ureter was further trimmed. A new ureteral anastomosis was performed to the augmented portion of the urinary bladder over a 6‐French, 12 cm double‐J stent using 6‐0 PDS. A suprapubic catheter was placed. Serum creatinine returned to normal by postoperative Day 3 to 0.93 with an eGFR of 60.9 mL/min/1.73m^2^. She was discharged on postoperative Day 9. No bacterial or fungal growth was noted on final culture of the excised pseudoaneurysm. The suprapubic catheter was removed 3 weeks after surgery followed by removal of the double J stent. One year after the procedure her serum creatinine was noted to be 1.3 mg/dL (Table 1). Six years post‐autotransplantation the patient remains off dialysis with a serum creatinine of 2.08 mg/dL (eGFR 34 mL/min/1.73m^2^).

**Table 1:** Timeline of events.

March 14, 2020	Initial transplant
March 29, 2020	Readmission: Urine culture positive for ESBL *E. coli*. Blood culture positive for ESBL *E. coli*. Vancomycin and cefepime started
March 31, 2020	Perinephric abscess drained and positive for *E. coli*. Transitioned to meropenem until 4/15
April 13, 2020	IR drain removed
April 24, 2020	Readmission #2: Blood cultures positive for ESBL *E. coli*. Urine culture positive for *Enterococcus avium* and *Klebsiella pneumoniae*. One dose of vancomycin given then meropenem restarted.
May 22, 2020	Ureteral stent removal
May 28, 2020	Readmission #3: Blood and urine cultures were both negative at this time. Patient continued on meropenem.
June 2, 2020	Initial takeback: Abdomen left open because of coagulopathy after completion of autotransplantation.
June 4, 2020	Second takeback: Found to have ischemia at ureter anastomosis requiring revision. Patient able to be closed.
June 13, 2020	Discharge

## 3. Discussion

We present the first reported case of successful renal allograft salvage with autotransplantation for the management of a suspected infected transplant renal arterial pseudoaneurysm in a pediatric patient. Anastomotic pseudoaneurysms are an uncommon complication in renal transplant recipients. Causes include infectious (bacterial or fungal), technical complications, immune factors, degeneration of saphenous vein interposition grafts, or idiopathic causes [2, 5]. Presentations of renal transplant pseudoaneurysms vary from incidental discovery on imaging to decreased renal function, pain, or even leg ischemia if the pseudoaneurysm progresses. In our case, the discovery was incidental in the workup of fever with recurrent urinary infections.

There are no clear guidelines on the indications for repair of renal transplant pseudoaneurysms. Prior literature supports intervention if the pseudoaneurysm is > 2.5 cm, there is continued growth, or if there is an increased risk of rupture such as in the case of infectious pseudoaneurysm [6]. Definitive management can be achieved with transplant nephrectomy. In this pediatric recipient (PRA 96%), our goal was to preserve her functioning allograft since finding a suitable allograft from a deceased or living donor (as she was adopted from overseas) in the future would be challenging.

Osler originally defined mycotic aneurysms in the setting of endocarditis, where bacterial embolization led to infection of the arterial wall with dilation. The term, however, has grown to have variable meaning within the literature with some reserving mycotic to indicate fungal infection whereas others use it as a catch‐all term for infectious aneurysms [7]. Reports of infectious renal aneurysms in transplant literature have described the formation of these infectious aneurysms by two mechanisms. The first is noted transiently after bacteremia and the second mechanism is after localized infection with an abscess occurring near the iliac vessels. Both mechanisms may have contributed to the pathology in our patient. Definitive confirmation of infection within the pseudoaneurysm can be a clinical challenge because of the need for antibiotics prior to tissue culture. Additional testing such as 16S rRNA PCR can be utilized to improve pathogen detection in this setting. Our patient had been on meropenem for 39 days prior to excision of the pseudoaneurysm which may have sterilized the collection. Given the recurrent bacteremia and proximity of the previously infected perinephric fluid collection, we suspect infectious pseudoaneurysm to be the most likely cause despite the negative cultures. However, the lack of 16S rRNA evaluation or positive wound cultures is a limitation of this study.

A review of reported infectious or mycotic renal transplant pseudoaneurysms identified 36 reported cases of bacterial pseudoaneurysms of which 15 grafts were salvaged through either watchful waiting, stenting, or surgical repair [2]. Fujikata et al. reported a patient who developed a 4 cm suspected infectious pseudoaneurysm that developed after MRSA bacteremia. The infection was managed with vancomycin and the pseudoaneurysm was followed for 38 months without rupture or change in size [8]. However, this report is atypical and multiple cases exist of infectious pseudoaneurysm rupture [6, 9]. In immunocompetent patients, infectious pseudoaneurysms may be safely managed by endovascular intervention and suppressive antibiotics. However, in patients receiving immunosuppression, there remains concern regarding long‐term stability and recurrence of infection with endovascular management. Haijie et al. reported two cases of infectious pseudoaneurysms treated with endovascular stenting that subsequently developed anastomotic rupture, hemorrhage, and subsequent graft removal [9]. They concluded that stenting of renal transplant infected pseudoaneurysms was insufficient treatment. This aligns with Caño‐Velasco et al. who created an algorithm for the management of infectious renal artery pseudoaneurysms. They recommend endovascular control for temporary first‐line management in unstable patients, with definitive surgical repair or excision once the patient is stabilized [10]. Conversely, Anders et al. reported success with coil embolization, stenting, and antibiotics for a patient with a pseudoaneurysm and recurrent UTI [2]. Of note, their patient never had an abscess or bacteremia, so it is possible that the pseudoaneurysm was not truly due to infectious causes.

Surgical repair can be performed either by excision of the aneurysm and patch coverage or excision with an interposition graft. Interposition grafts previously described in the literature include synthetic grafts, arterial allografts, and saphenous vein autografts [5]. Synthetic grafts have been successfully used by Bracale et al. for autotransplant in the setting of a noninfectious pseudoaneurysm [6]. Interestingly, Berglund et al. reported the attempted management of an infectious renal transplant arterial pseudoaneurysm by excision and polytetrafluoroethylene patch coverage. The patch subsequently became infected and then required excision with donor iliac artery interposition graft and renal autotransplantation [11]. Blood group matched arterial allografts from tissue banks represent an ideal material in this circumstance as there is a low risk of infection. For our case, we chose to use deceased donor arterial allograft because of the risk of placement into a potentially infected field. For the arterial anastomosis in autotransplantation, options include anastomosis to the internal iliac artery, common iliac artery, interposition graft, or contralateral artery. We chose to directly anastomose the renal transplant artery to the arterial interposition graft given the positive experience reported in the literature. Additionally, we wanted to preserve the contralateral iliac fossa for future transplant in this pediatric recipient.

Our review of the literature identified eight prior reports of autotransplantation (Table 2) as a management strategy for renal allograft preservation, all with good outcomes [6, 11–16]. Prior indications for autotransplants of allografts included pseudoaneurysms, true aneurysms, infected synthetic patches, iliac stent migration, and movement of an allograft outside of the field of pelvic radiation. Though some noted delayed graft function or acute tubular necrosis, all grafts regained appropriate function. It is important to note that surgical repair has its own risks because of increased adhesions in the setting of infection. In our case we were able to complete the anastomoses; however, we had to leave the abdomen temporarily packed for coagulopathy correction after the surgery. Campbell et al. also noted significant difficulty with dissection, resulting in the autotransplant being delayed for 24 h in order to allow for hemodynamic and coagulopathy stabilization [12]. When choosing to perform an autotransplant we recommend being prepared for coagulopathy with blood product availability, coordinating with anesthesia prior to the surgery, and having access to rapid transfusion devices.

**Table 2:** Renal allograft excision and autotransplantation: review of literature.

Authors	Patient age	Indication for intervention	Management	Culture	Outcome
Hanlon et al. [5]	45F	4.2 cm aneurysm of renal artery to saphenous vein interposition graft to IIA	Excision of IIA. Interposition iliac artery homograft to CIA	Not suspected	eGFR 51 mL/min at 4 months
Bracale et al. [6]	39F	5.5 cm Pseudoaneurysm	Excision with polyester interposition graft. Autotransplant to hypogastric artery	Negative	Alive 39 months
Berglund et al. [11]	27 M	4 cm pseudoaneurysm previously repaired by excision and PTFE patch. Subsequent infection of PTFE patch.	Resection of EIA and PTFE patch with interposition vascular allograft and autotransplant	Positive for *Pseudomonas aeruginosa*	Kidney function preserved without further infection at 24 months
Campbell et al. [12]	^∗∗∗^	Pseudoaneurysm	Excision of pseudoaneurysm with autotransplantation	^∗∗∗^	^∗∗∗^
Casingal et al. [13]	43 M	Stent migration into EIA, covering transplant renal artery	Excision of EIA and autotransplant to CIA	Not suspected	Postoperative ATN. Returned and maintained baseline Cr of 2.2 at 15 months.
DeRoover et al. [14]	48F	Removal from field of pelvic irradiation for vulvar cancer	Autotransplantation to contralateral EIA	Not suspected	Cr 1.1 postoperatively
Chandak et al. [15]	40 M	3.2 cm true aneurysm (concern for mycotic pseudoaneurysm on imaging)	Excision of EIA with saphenous vein interposition graft and primary repair of EIV. Autotransplantation to contralateral IIA.	Negative	Cr 1.3 after 19 days
Mezzetto et al. [16]	40 M	5.2 cm aneurysm of renal artery	Autotransplant, excision renal artery pseudoaneurysm, anastomosis renal artery	Not suspected	Delayed graft function, normal Cr at 3 months

Note:  ^∗∗∗^ indicates inability to view the original article.

We conclude that selective renal allografts with suspected infectious pseudoaneurysms at the arterial anastomosis may be salvaged successfully with transplant nephrectomy and reimplantation after excision of the aneurysmal artery. Arterial donor interposition grafts are an ideal material in this setting because of their inherent ability to limit infectious risk. This allograft salvage strategy may be especially beneficial in highly sensitized children who may need multiple transplants during their lifetime. Although technically challenging and requiring adequate blood product support, autotransplantation is feasible and should be considered as an option for managing this rare postrenal transplant complication.

## Funding

No funding was received for this manuscript.

## Conflicts of Interest

The authors declare no conflicts of interest.

## Data Availability

The data that support the findings of this study are available from the corresponding author upon reasonable request.
